# Trans Fat Intake and Its Dietary Sources in General Populations Worldwide: A Systematic Review

**DOI:** 10.3390/nu9080840

**Published:** 2017-08-05

**Authors:** Anne J. Wanders, Peter L. Zock, Ingeborg A. Brouwer

**Affiliations:** 1 Unilever R & D Vlaardingen, 3133 AT Vlaardingen, The Netherlands; Peter.Zock@unilever.com; 2 Department of Health Sciences, Faculty of Earth & Life Sciences, Vrije Universiteit Amsterdam, Amsterdam Public Health Research Institute, 1081 HV Amsterdam, The Netherlands; Ingeborg.Brouwer@VU.nl

**Keywords:** trans fatty acid, industrial trans fat, partially hydrogenated vegetable oils, ruminant trans fat, dietary sources, national dietary survey, review

## Abstract

After the discovery that trans fat increases the risk of coronary heart disease, trans fat content of foods have considerably changed. The aim of this study was to systematically review available data on intakes of trans fat and its dietary sources in general populations worldwide. Data from national dietary surveys and population studies published from 1995 onward were searched via Scopus and websites of national public health institutes. Relevant data from 29 countries were identified. The most up to date estimates of total trans fat intake ranged from 0.3 to 4.2 percent of total energy intake (En%) across countries. Seven countries had trans fat intakes higher than the World Health Organization recommendation of 1 En%. In 16 out of 21 countries with data on dietary sources, intakes of trans fat from animal sources were higher than that from industrial sources. Time trend data from 20 countries showed substantial declines in industrial trans fat intake since 1995. In conclusion, nowadays, in the majority of countries for which data are available, average trans fat intake is lower than the recommended maximum intake of 1 En%, with intakes from animal sources being higher than from industrial sources. In the past 20 years, substantial reductions in industrial trans fat have been achieved in many countries.

## 1. Introduction

Trans fats are found in foods originating from ruminant animals, such as cows and sheep, and are found in foods containing partially hydrogenated vegetable oils (PHVO). Animal trans fat levels can comprise up to 6% of a product’s fat content in ruminant foods, and industrial trans fat levels can comprise up to 60% of a product's fat content in foods containing PHVO. The discovery of adverse effects on the blood cholesterol profile and the increased risk of coronary heart disease of industrial trans fat [1,2,3,4,5] have led to public health recommendations to lower total trans fat intake to below 1% of total energy intake (En%), primarily by the removal of industrial trans fat [6]. Subsequent voluntary initiatives, trans fat labelling measures and regulatory limits have led to reductions in industrial trans fat, although not in all foods and countries [7,8].

Earlier studies showed that over the past two decades, both the voluntary and regulatory measures to lower industrial trans fat content of foods have resulted in significant reductions in global trans fat intakes [9,10,11,12]. In 1995, the TRANSFAIR study showed that in most Western European countries, the intakes of total trans fat were already below 1 En%. This study also showed that in the majority of the countries, trans fat primarily came from animal sources [13]. However, across countries, large differences exist in the intakes of trans fat. In 2010, estimated intakes varied from 0.2 to 6.5 En% worldwide [10]. At present, many countries actively work on measures to further lower industrial trans fat levels in foods [7,14,15].

Up-to-date data on global trans fat intakes, and its dietary sources are relevant for the development of public health measures and to monitor their effectiveness. Therefore, this study aimed to provide an up-to-date review of intakes of trans fat and its dietary sources in general populations worldwide. A secondary aim was to quantify trans fat and saturated fat levels in biscuits, a food that can nowadays still be relatively high in trans fat, to serve as a marker for the presence of industrial trans fat in countries for which no population dietary intake data are available [16].

## 2. Materials and Methods

### 2.1. Search Strategy

We conducted a broad, systematic search strategy to identify dietary intake studies. Peer reviewed papers, reports and grey literature on trans fat intake in countries and regions in any language published after 1995 were considered for inclusion. First, Scopus was searched through 29 May 2017 with the following search terms: “diet” AND “survey” AND (“trans fat” or “trans fatty acids”). In addition, all references reporting trans fat intake from two recent publications on global fat intake were screened [10,17]. Additionally, for each national survey that was identified, earlier and later editions were hand searched through Scopus and Google, and national intake data were checked via the websites of national public health institutes (Supplemental Figure S1). Methods of the analysis and inclusion criteria were not documented in a registered review protocol.

We determined eligibility of all publications and reports based on the following inclusion criteria: (1) national survey or population-based study; (2) trans fat intake measured by food composition data or new analytical data; (3) published after 1995; (4) data from the general adult population; and (5) reporting either the calendar year of the dietary survey or of the food composition data. For countries with multiple datasets, we included all data published after 1995.

For our secondary research question aiming to quantify trans fat and saturated fat levels in biscuits, we conducted a search on Scopus with the elements: “trans fat” AND “composition”. All identified publications were screened to determine eligibility based on the following inclusion criteria: (1) random sampling of biscuits or cookies; (2) new analytical data measured by gas chromatography; (3) published after 2005; (4) reporting both trans fat and saturated fat composition.

### 2.2. Data Extraction and Analysis

From publications or reports that met the inclusion criteria, one investigator extracted the following information: country, type and year of dietary intake survey, type and year of food composition data, age group (19–64 years-or the nearest age), sample size, mean, standard deviation (SD), and 95th percentile of total trans fat intake (g/day and En%), trans fat intake from animal sources (%) and industrial sources (%), and reported local measures to lower trans fat intake (voluntary, mandatory, and year).

Where total trans fat intake was reported both as fatty acids and as fat, we used fatty acids. Where trans fat intake was not reported in either g/day or En%, intakes in these units were calculated using reported energy intake and the energy content of dietary fat of 9 kcal/g. Where data were reported for subgroups (for example by gender), a weighted mean for the total population was calculated by weighing the mean intake of each subgroup by the number of the subjects in this subgroup.

“Animal sources” of trans fat were defined as milk, cheese, eggs, meat, fish, butter, and “industrial sources” of trans fat were defined as oils and fats, biscuits, pizza, grains, seeds, nuts, chocolate, soups, savory snacks, meals, and restaurant foods. In specific publications and reports, clustered food items could contain both animal and industrial sources of trans fat. We regarded these food items an industrial source of trans fat. Examples were butter being clustered in a “fats and oils” food group, and ice-cream being categorized in a “sweet snacks” food group.

The included data were assessed and scored on quality using four criteria: type of survey data (maximum score of 3), dietary assessment method (maximum score of 3), type of food composition data (maximum score of 3), and sample size (maximum score of 2) [18] (Supplemental Table S1). The total attainable score of a study ranged between 4 and 11. Based on the score, three quality categories were defined: high quality (score of 10–11), medium quality (score of 8–9), low quality (score of 4–7).

For dietary intake data, mean population intakes per country were compared to the recommended intake level of less than 1 En% as determined by the Food and Agriculture Organization (FAO)/World Health Organization (WHO) [6]. For biscuit data, mean trans fat levels were compared to the Danish regulatory limit of less than 2 g per 100 g total fat.

## 3. Results

The systematic search in Scopus and in references from available publications on global fat intake [10,17] identified 265 publications. After screening, 27 publications were judged eligible for inclusion in the review. Additional hand searching resulted in 16 eligible publications. A flow-chart and reasons for exclusion are given in Supplemental Figure S1. The 43 eligible publications included 64 datasets representing 29 countries (Table 1). One study representing trans fat intakes in Costa Rica [19], was exempted from exclusion. This study should have been excluded as it reported data for adolescents, and not adults. It was decided to be included in the study in the review because it reported repeated measurements of trans fat intakes over time, which was considered to be of sufficient importance to deviate from the inclusion criteria.

**Figure 1:**
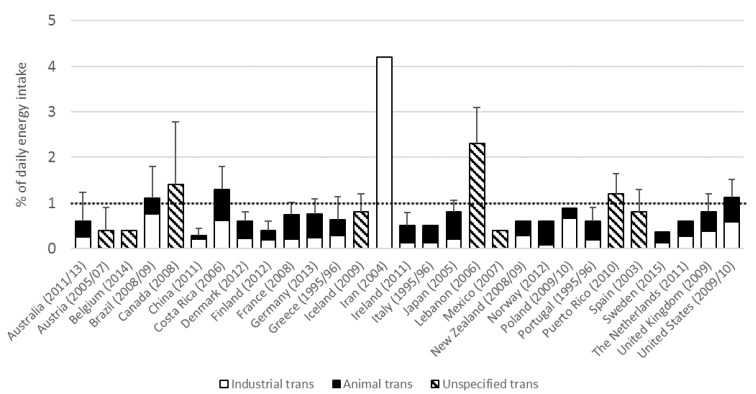
Mean (Standard Deviation) intakes of trans fat and its sources in populations from 29 countries. Data represent estimates of population total trans fat intake reported by year of food composition data. The dotted line represents the maximal recommended intake level of trans fat [6].

The most up-to-date estimates of population total trans fat intake ranged from 0.3 to 4.2 percent of energy intake (En%). In 22 out of 29 (76%) countries mean trans fat intake was below the WHO recommendation of 1 En% (Figure 1). At the time of the survey, Brazil (2008/09), Canada (2008), Costa Rica (2006), Iran (2004), Lebanon (2006), Puerto Rico (2010) and USA (2009/10) reported trans fat intakes above 1 En%. In 16 out of 21 (76%) countries with data on trans fat sources, mean intakes of animal trans fat were higher than that of industrial trans fat. Industrial trans fat intake was higher in Brazil (2008/09), China (2011), Iran (2004), Poland (2009/10) and USA (2009/10).

**Figure 2:**
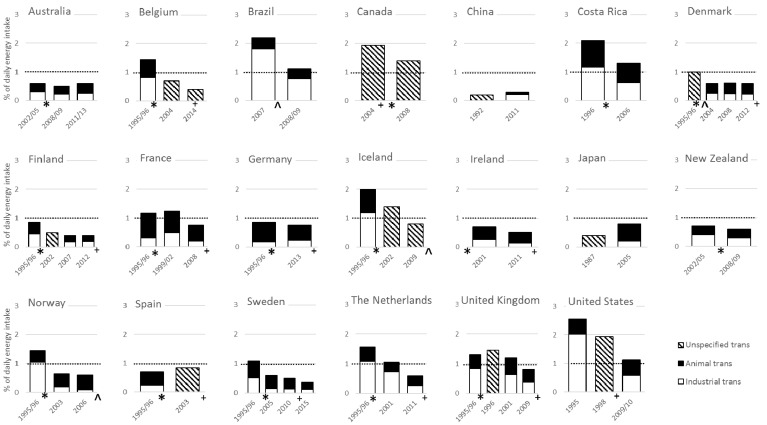
Time trends of intakes of trans fat and its sources in populations from 20 countries. Data reported by year of food composition data. Symbols on the time-axis refer to the year of the introduction of voluntary reductions (*), mandatory labeling (^+^), or mandatory limits (^) to lower trans fat intake. The dotted lines represent the maximal recommended intake level of trans fat [6].

For 20 countries we found multiple surveys over time so that time trends could be made (Figure 2). These time trends showed substantial declines in trans fat intake over the past two decades. In seven countries, mean intakes decreased from above 1 En% to below 1 En%. Nine countries had trans fat intakes that were already equal or below 1 En% at the time of the first survey, and in four countries trans fat intake did not decrease to below 1 En%. The largest reductions were seen in industrial trans fat intake, although in several countries also trans fat from animal sources reduced. The introduction of voluntary or mandatory measures to lower trans fat in foods were associated with reductions in trans fat intakes in many countries.

**Figure 3:**
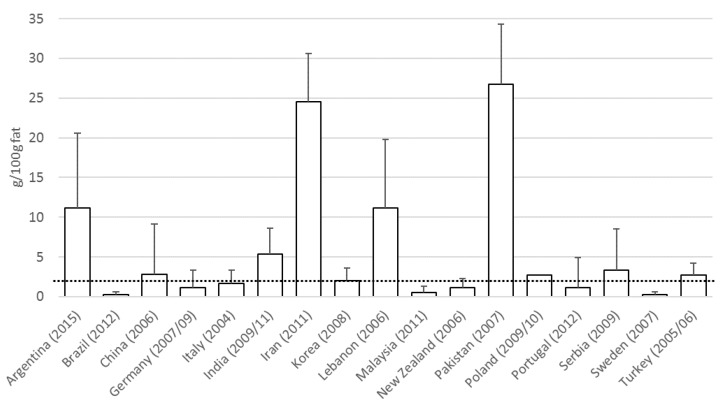
Mean (Standard Deviation) trans fat content of biscuits in 17 countries. Data reported by year of sampling. The dotted line represents the Danish regulatory limit for foods of less than 2 g trans fat per 100 g total fat.

The search on trans and saturated fat content of biscuits resulted in 20 publications and reports representing 17 countries (Supplemental Table S2). Of these countries, seven did not have data on population total trans fat intake. At the time of the surveys in Brazil (2012), Germany (2007/09), Italy (2004), Malaysia (2011), New Zealand (2006), Portugal (2012) and Sweden (2007) the trans fat content of biscuits was below the Danish limit of 2 g per 100 g fat. In Argentina (2015), China (2006), India (2009/11), Iran (2011), Korea (2008), Lebanon (2006), Pakistan (2007), Poland (2009/10), Serbia (2009) and Turkey (2005/06) the reported trans fat content of biscuits was higher, up to 26.7 g/100 g fat (Figure 3).

Time trends of the solid fat composition of biscuits could be made for Brazil, Korea, Malaysia, Serbia and Sweden (Supplemental Figure S2). In Brazil, Korea, Malaysia and Serbia, the decrease in trans fat content in biscuits was accompanied by equal or higher content of solid fat (sum of saturated fat and trans fat), whereas in Sweden the solid fat content of biscuits decreased.

## 4. Discussion

The data summarized in this systematic review show that for 22 out of 29 countries, the intake of total trans fat is currently below the recommended maximum intake of 1 En%. Like earlier studies from Craig-Smith and Micha [9,10], we also showed that total trans fat intakes have been decreasing over the past decades. This review is the first to distinguish trans fat intake from animal and industrial sources.

Several limitations of our analysis should be considered when interpreting the results. The first is heterogeneity of the trans fat intake data with respect to different sampling and dietary assessment methods within and across countries. For example, not all datasets provide national representative food survey data, as we also included household intake surveys and intake data from large observational studies in general populations. As a result, differences in reported trans fat intakes between and within countries may be partly due to differences in the data collection methods.

A second limitation is the limited reliability of data on trans fat content of foods in local food composition tables. Determination of trans fat content is often not included in routine fatty acid analyses of foods. Also, trans fat consists of different isomers, which occur in different levels in different foods. As a result, food composition tables tend to be more incomplete for trans fat than for other nutrients. This may increase the number of borrowed or estimated data which can lead to larger bias in estimations of true intakes. Also, data on trans fat content of foods can be outdated because of changing food compositions (reduced trans content), which can contribute to overestimation of true trans fat intakes. It was not possible to evaluate the quality of food composition databases and trans fat isomers included in the analyses in different surveys, because the reported information was in many cases too limited. For several national surveys, analytical data from market basket studies on trans fat content were newly generated. As such data represent actual average population intakes, these data can be regarded as the most reliable.

Third, data on trans fat intake are limited or not available for many countries in less developed regions, in particular Asia, Africa and the Middle East. Also, some more developed countries such as Sweden stopped reporting trans fat in their national dietary surveys in 2010, reasons may be that trans fat intake was below the recommended maximum intake of 1 En% and therefore not high priority for public health [61] or that data quality was considered insufficient. Trans fat intakes reported in this review for specific countries may therefore not be representative for other countries.

The last limitation is the imperfect distinction between industrial, animal and ruminant trans fat sources. Ruminant trans fat mainly comes from dairy, beef, and lamb, but non-ruminant animal foods such as pork, chicken and fish can also contain trans fat [62]. The source of this trans fat is unclear, but it may come from animal feed made with industrial trans fat. In this review we reported total animal trans fat, because in most dietary surveys no distinction was made between ruminant and non-ruminant animal food groups. The reporting of animal trans fat sources may lead to a minor overestimation of ruminant trans fat intakes. For example, in the 2011 survey from the Netherlands, 0.32 En% of the total trans fat intake came from ruminant animal sources and 0.01 En% from non-ruminant animal sources. In addition, in case a food group in a dietary survey contained both animal and industrial foods (e.g., the food group ”fats and oils” comprising both butter and margarines), we considered this food group an industrial trans fat source. This may underestimate animal trans fat and overestimate industrial trans fat intakes. For example, in one third of the datasets trans fat intake from butter was not separately reported. From available data, we calculated that per country the average trans fat intake from butter was 0.07 En%. As a result, industrial trans fat intake may have been overestimated. Likewise, the trans fat source in biscuits could either be industrial sources or butter. Butter may comprise trans fat levels up to 6% of total fat. In an extreme scenario assuming that all biscuits containing trans fat levels up to 6% of total fat are made from butter, in four countries, Argentina (2015), Iran (2011), Lebanon (2006) and Pakistan (2007) biscuits must have high industrial trans fat levels.

Overall, this review showed that in almost all countries included in this review, the intake of trans fat has substantially decreased over the past 20 years. In 22 out of 29 countries (76%) the average intake is now well below the recommended maximum intake of 1 En%. The reduction in trans fat intake can be primarily attributed to the reduction of the use of industrial trans fat. Apparently, voluntary self-regulation by the industry, trans fat labelling, and local or national regulatory measures on use of PHVO or trans fat in the food supply chain [7,8,63] have successfully lowered industrial trans fat intake in these countries. However, a decline of the use of trans fat in foods has not yet happened everywhere. In many countries, including USA and the European Union, statutory measures to reduce trans fat intake do not yet exist, or are only recently in place or effective [7,15]. Our review of the composition of fats in biscuits showed that in several countries foods can still contain high levels of trans fat. Assuming that biscuits are a marker for the presence of industrial trans fat in other foods [16], industrial trans fat intakes in these countries may still be substantial. It should also be realized that even if average trans fat intakes in a country are low, specific subgroups with a preference for certain foods (such as low income groups preferring specific brands of biscuits) may consume trans fat in amounts that significantly increase their risk of coronary heart disease [11,64].

Concerns have been voiced that food manufacturers may replace trans fat in foods with saturated fats, to maintain the required or preferred solid fat content (saturated fat plus trans fat) in the food [65,66]. Such replacements are not in agreement with public health recommendations to lower trans fat intake and to replace saturated fat intake with polyunsaturated fat to decrease the risk of coronary heart disease [6]. However, studies in North America show that over the past 10–15 years, most supermarket and restaurant foods decreased trans fat content without a concomitant increase in saturated fat [67,68]. Nevertheless, this was not the case for all foods in all countries [66]. This was also reflected in our findings that in Sweden the solid fat content of biscuits decreased, whereas the solid fat content of biscuits in Brazil, Korea, Malaysia, and Serbia remained stable or increased. For solid foods such as biscuits, technical innovations are required to preserve texture properties while replacing trans fat with unsaturated fat, instead of saturated fat [65]. The present review suggests that thus far, not all food manufacturers have applied these technical innovations.

This review also indicated that with the considerable reductions in the intake of industrial trans fat, the primary dietary source of trans fat is now animal foods. Industrial and animal trans fats are likely to have comparably adverse effects on the blood cholesterol profile at similar intake levels [69,70]. However, because intakes of animal trans fats are generally well below 1 En%, their impact on the blood cholesterol profile and coronary heart disease risk in the population is limited. The intake of ruminant trans fat is therefore not seen as a major dietary problem for public health [71]. The most important sources of animal trans fat are full-fat dairy products and high-fat meats. Current food-based dietary guidelines advise to reduce saturated fat intake by limiting the intake of full-fat dairy products and high-fat meats. Adhering to these guidelines will also reduce the intake of animal trans fat [72,73,74]. While many countries now have average trans fat intakes below 1 En%, recent research suggests that trans fat intakes at dosages around 1 En% may still adversely affect outcomes other than cardiovascular risk, such as reproductive health [75,76]. However, more research is needed to confirm benefits of further reducing trans fat intakes.

## 5. Conclusions

In the past 20 years the total intake of trans fat substantially decreased. In the majority of countries for which data are available, average trans fat intake is nowadays lower than the recommended maximum intake of 1 En%, with intakes from animal sources being higher than from industrial sources. Both voluntary and regulatory measures to reduce the intake of industrial trans fat have resulted in substantial reductions in many countries.

## Figures and Tables

**Table 1:** Mean intakes of trans fat and its dietary sources in populations from 29 countries.

Country [Reference]	Survey Type	Survey Method	Survey Year	Food Composition Data Type	Year Food Composition Data	Age	Sample Size	Mean Trans Fat Intake	Mean Trans Fat Intake	SD Trans Fat Intake	95th p Trans Fat Intake	Animal Trans	Industrial Trans	Study Quality ^#^	Trans Fat Lowering Measures (Year)
						Year	Number	g/day	En%	En%	En%	%	%		
Australia [20]	National	24hr recall	1995	Market basket	2002/2005	17+	~11,000	1.5	0.6		1.2	51	49	Medium	Voluntary reduction (2007)
Australia [20]	National	24hr recall	1995	Market basket	2008/2009	17+	~11,000	1.3	0.5		1.1	55	45	Medium	
Australia [21]	National	24hr recall	2011/12	Food database	2011/2013	19+	~9000	1.4	0.6	0.6		58	42	Medium	
Austria [22]	Local	24hr recall	-	Market basket	2005/2007	14–36	2989	1.0	0.4	0.5	1.4			Medium	Mandatory limit (2009) ^6^
Belgium [13]	Local	3 d record	1991/92	Market basket	1995/1996	18–65	492	4.1	1.4	0.5	2.1 ^3^	44	57	Medium	
Belgium [23]	National	2 × 24hr recall	2004	Food database	2004	15–64	3252	1.9	0.7		1.3			Medium	^6^
Belgium [24]	National	2 × 24hr recall	2014/15	Food database	2014	15–64	3138	0.9	0.4		0.6			Medium	
Brazil [25]	Local	24hr recall	2003	Food database ^2^	2007	20–59	713	4.5	2.2	2.7		18	82	Low	Mandatory limit (2007)
Brazil [26,27]	National	2 d record	2008/09	Food database	2008/2009	10+	32,749	2.4	1.1	0.7		31	69	Medium	
Canada [28]	National	24hr recall	2004	Food database	2004	19+	19,053	4.7	1.9	2.8				Medium	Mandatory labeling (2005), voluntary reduction (2007)
Canada [28]	National	24hr recall	2004	Market basket	2008	19+	19,053	3.4	1.4	1.4				Medium	
China [29]	Local	4 × 24hr recall	1997/99	Food database	1992	40–59	839	0.5	0.2	0.4				Low	
China [30]	Local	3 × 24hr recall	2011	Market basket	2011	>18	4424	0.6	0.3	0.2	0.6	27	73	Medium	
Costa Rica [19]	Local	3 d record	1996	Food database	1996	12–17	275	4.5	2.1	0.9		45	55	Medium	Voluntary reduction (1996)
Costa Rica [19]	Local	3 d record	2006	Food database	2006	12–17	133	2.8	1.3	0.5		52	48	Medium	
Denmark [13]	National	7 d record	1995	Market basket	1995/1996	1–80	3000	2.6	1.0	0.5	1.5 ^3^			High	Mandatory limit (2003) ^6^
Denmark [31]	National	7 d record	2000/02	Food database	2004	18–75	3151	1.5	0.6	0.2	0.8 ^3^	56	43	High	
Denmark [32]	National	7 d record	2003/08	Food database	2008	18–75	3354	1.4	0.6	0.2	0.8 ^3^	61	41	High	
Denmark [33]	National	7 d record	2011/13	Food database	2012	18–75	3016	1.5	0.6	0.2	0.8 ^3^	62	38	High	
Finland [13]	National	3 d record	1992	Market basket	1995/1996	25–64	1861	2.1	0.9	0.3	1.2 ^3^	46	53	High	^6^
Finland [34]	National	48hr recall	2002	Food database	2002	25–64	2007	1.0	0.5	0.2				Medium	
Finland [35]	National	48hr recall	2007	Food database	2007	25–64	1576	0.8	0.4	0.2		55	45	Medium	
Finland [36]	National	48hr recall	2012	Food database	2012	25–64	1295	1.0	0.4	0.2		53	47	Medium	
France [13]	National	7 d record	1993/94	Market basket	1995/1996	15–65	1500	2.3	1.2	0.3	1.6 ^3^	75	26	High	^6^
France [37]	National	7 d record	1998/99	Market basket	1999/2002	15+	1985	3.0	1.2	0.4	2.0	60	40	High	
France [38]	National	7 d record	2006/07	Food database	2008	18–79	1918	1.8	0.8	0.3	1.2	72	28	High	
Germany [13]	Local	diet history	1991	Market basket	1995/1996	18–80	1897	2.1	0.9	0.2	1.1 ^3^	79	21	Medium	Voluntary reduction (2012) ^6^
Germany [39]	National	4 w diet history	2005/06	Food database	2013	14–80	15,371	1.9	0.8	0.3	1.3	70	30	Medium	
Greece [13]	Local	24hr recall	1995	Market basket	1995/1996	23–64	248	1.4	0.6	0.5	1.2 ^3^	55	46	Low	^6^
Iceland [13]	National	diet history	1990	Market basket	1995/1996	15–80	1240	5.4	2.0	0.6	2.7 ^3^	40	60	High	Mandatory limit (2011)
Iceland [40]	National	24hr recall	2002	Food database	2002	15–80	1242	3.5	1.4	0.9				Medium	
Iceland [41]	National	2 × 24hr recall	2010/11	Food database	2009	18–80	1312	1.8	0.8	0.4				Medium	
Iran [42]	National	3 × hh 24hr recall	2001/03	Market basket	2004	all	35,924	12.3	4.2			- ^1^	100	Medium	Mandatory limit (2004)
Ireland [43]	National	4 d record	1997/99	Food database	2001	18–64	1097	1.9	0.7	0.3		62	38	High	^6^
Ireland [43]	National	4 d semi-w record	2008/10	Food database	2011	18–64	889	1.3	0.5	0.3		74	26	Medium	
Italy [13]	National	7 d hh record	1980/84	Market basket	1995/1996	1–80	10,000	1.6	0.5			76	24	Medium	^6^
Japan [29]	Local	4 × 24hr recall	1997/99	Food database	1987	40–59	1145	0.9	0.4	0.3				Medium	
Japan [44]	Local	16 d semi-w record	2002/03	Food database	2005	30–69	225	1.7	0.8	0.3	1.9 ^4^	74	26	Medium	
Lebanon [45]	Local	FFQ	2009/11	Food database ^2^	2006	19–70	657	6.1	2.3	0.8				Low	
Mexico [46]	National	FFQ	2006	Food database	2007	20–60	16,366	0.5	0.4	0.5				Medium	
New Zealand [20]	National	24hr recall	1997	Market basket	2002/2005	15+	4636	1.9	0.7		1.3	43	57	Medium	Voluntary reduction (2007)
New Zealand [20]	National	24hr recall	1997	Market basket	2008/2009	15+	4636	1.6	0.6		1.2	52	48	Medium	
Norway [13]	National	FFQ	1993/94	Market basket	1995/1996	16–79	3144	4.0 ^6^	1.4	0.5	2.2 ^3^	28	72	Medium	Mandatory limit (2014)
Norway [47]	National	FFQ	1997	Food database	2003	16–79	2672	1.6	0.6			71	29	Medium	
Norway [48]	National	hh record	2012	Food database	2006	All	4125 hh	1.6	0.6			87	13	Low	
Poland [49]	National	1 m hh record	2009/10	Market basket	2009/2010	all	N/A	2.0	0.9			25	75	Medium	^6^
Portugal [13]	National	24hr recall	1988/89	Market basket	1995/1996	38	78 men	1.6	0.6	0.3	1.1 ^3^	69	31	Medium	
Puerto Rico [50]	Local	6 d record	2012	Food database	2010	21+	92	2.5	1.2	0.4				Medium	Mandatory limit (2007)
Spain [13]	National	7 d hh record	1991	Market basket	1995/1996	0–70	21,555	2.1	0.7			64	35	Medium	^6^
Spain [51]	Local	FFQ	2000	Food database	2003	18–77	516	1.9	0.8	0.5				Low	
Sweden [13]	National	7 d record	1989	Market basket	1995/1996	1–74	3000	2.6	1.1	0.5	1.6 ^3^	53	47	High	^6^
Sweden [52]	National	Product/trade data	2003	Market basket	2005	all	N/A	1.9	0.6			73	27	Low	
Sweden [52]	National	Product/trade data	2007	Market basket	2010	all	N/A	1.7	0.5			73	27	Low	
Sweden [53]	National	Product/trade data	2013	Market basket	2015	All	N/A	1.2	0.4			64	36	Low	
The Netherlands [13]	National	2 d record	1992	Market basket	1995/1996	1–92	6218	4.3	1.6	0.7	2.4 ^3^	29	70	High	^6^
The Netherlands [54]	National	2 × 24hr recall	2003	Food database	2001	19–30	750	2.8	1.1	0.5	1.5	30	70	Medium	
The Netherlands [55]	National	2 × 24hr recall	2007/10	Food database	2011	19–69	2106	1.5	0.6		0.9	56	44	Medium	
United Kingdom [13]	National	7 d hh record	1996	Market basket	1995/1996	0–75	7921	2.8	1.3			37	64	Medium	Voluntary reduction (2011) ^6^
United Kingdom [29]	Local	4 × 24hr recall	1997/99	Food database	1996	40–59	501	3.6	1.5	0.9				Low	
United Kingdom [56]	National	7 d record	2000/01	Food database	2001	19–64	1724	2.4	1.2	0.4	2.1 ^5^	47	53	High	
United Kingdom [57]	National	4 d record	2008/09	Food database	2009	19–64	434	1.6	0.8	0.4		53	47	Medium	
USA [58,59]	National	2 × 24hr recall	1994/96	Food database	1995	2+	11,258	5.3	2.6	2.1	3.2 ^3^	21	79	Medium	Mandatory labeling (2006), local bans
USA [29]	Local	4 × 24hr recall	1997/99	Food database	1998	40–59	2195	4.9	2.0	0.8				Medium	
USA [58,60]	National	2 × 24hr recall	2003/06	Food database	2009/2010	2+	~10,000	2.5	1.1	0.4		48	52	Medium	

Abbreviations: d = day; FFQ = food frequency questionnaire; hr = hour; hh = household; m = month; SD = standard deviation; w = week; 95th p = 95th percentile; ^#^ Study quality was based on four criteria: type of survey data, dietary assessment method, type of food composition data, and sample size (Supplemental Table S1). Based on the total attainable score three quality categories were defined: high quality (score 10–11), medium quality (score 8–9), low quality (score 4–7). ^1^ Animal trans fat was not assessed in Iran; ^2^ Food composition data originate from USA; ^3^ 90th percentile; ^4^ maximum; ^5^ 97.5th percentile; ^6^ Across Europe, voluntary measures were introduced as of 1995 and mandatory labeling of partially hydrogenated vegetable oils (PHVO) was introduced as of 2014.

## Supplementary Materials

The following are available online at www.mdpi.com/2072-6643/9/8/840/s1, Figure S1: PRISMA flow chart; Table S1: Criteria for evaluating scoring the data quality; Table S2: Mean trans fat and saturated fat composition of biscuits in 17 countries; Figure S2: Time trends of mean trans and saturated fat content of biscuits in 5 countries.
