# Supplementary material for: Trans Fat Intake and Its Dietary Sources in General Populations Worldwide: A Systematic Review
Source: Nutrients. 2017 Aug 5;9(8):840. doi: 10.3390/nu9080840 (PMC5579633; doi:10.3390/nu9080840)
Supplement: Supplementary file 1 [file nutrients-09-00840-s001.pdf]

Supplementary Data to:

Trans Fat Intake and Its Dietary Sources in General Populations Worldwide: A Systematic Review

Anne J. Wanders [1,*], Peter L. Zock [1] and Ingeborg A. Brouwer [2]

[1] Unilever R&D Vlaardingen, 3133 AT, Vlaardingen, The Netherlands; Peter.Zock@unilever.com
[2] Department of Health Sciences, Faculty of Earth & Life Sciences, Vrije Universiteit Amsterdam, Amsterdam Public Health Research Institute, 1081 HV, Amsterdam, The Netherlands; Ingeborg.Brouwer@VU.nl

* Correspondence: Anne.Wanders@unilever.com; Tel.: +31-104-605-348

This document contains the following supplemental data:

Supplemental Figure S1:	PRISMA flow chart
Supplemental Table S1:	Criteria for evaluating scoring the data quality
Supplemental Table S2:	Mean trans fat and saturated fat composition of biscuits in 17 countries
Supplemental Figure S2:	Time trends of mean trans and saturated fat content of biscuits in 5 countries

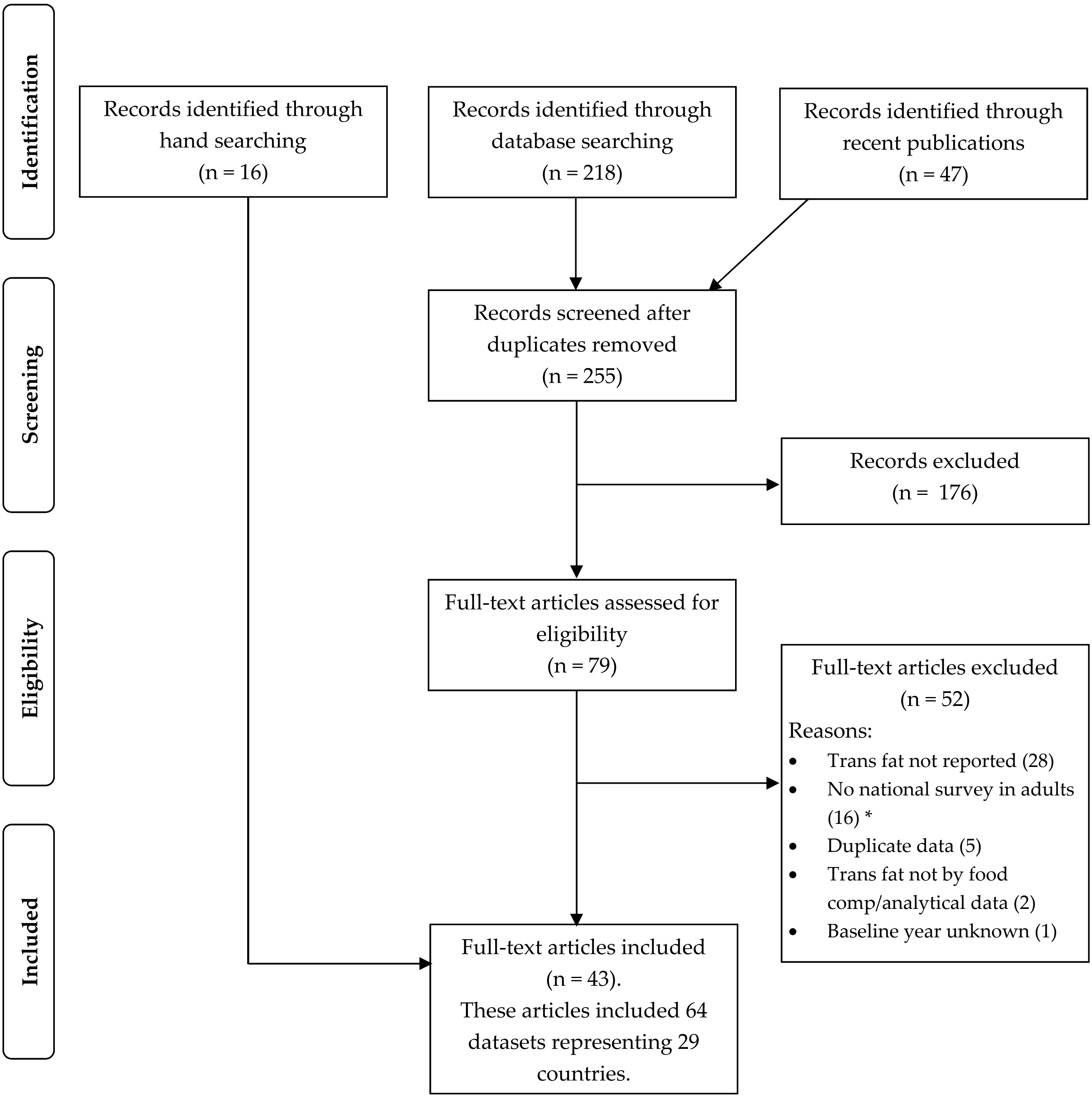

Figure S1. PRISMA flow chart.

* One study (Monge-Rojas et al., 2013) representing Costa Rica was exempted from exclusion. This study should have been excluded as it reported data for adolescents, and not adults. It was decided to include the study in the review because it reports repeated measurements of trans fat intakes over time, which was considered of sufficient importance to deviate from the inclusion criteria.

Table S1. Criteria for evaluating scoring the data quality.

Score	Type of survey data [1]	Dietary assessment method [2]	Type of food composition data [3]	Sample size [4]
1	Non-individual data	Single 24h recall or FFQ	Data borrowed from other country	<1000
2	Population-based study	Repeated 24h recall	Food composition database	>1000
3	National nutrition survey	Food record, minimum of 3d	Analytical data	
Maximum score	3	3	3	2

[1] National dietary surveys scored 3, as these are the preferred type of study to estimate the distribution of nutrient intake in a population. Population-based studies were scored 2, as these tend not to be nationally representative. Non-individual data, such as household budget surveys or product/trade data were scored 1, as they are of limited value in estimating the food intake of an individual [1-3].

[2] Weighed food record (minimal 3 days) methods were scored 3, as they are the highest-quality dietary assessment methods for estimating the usual intake of foods in individuals. Multiple 24h recalls were scored 3 as they increase the reliability and improves the dietary assessment. An FFQ or a single 24h recall scored 1, as on its own these methods have limitations in assessing usual intake [1-3].

[3] Analytical data generated from market basket studies were scored 3, as these represent up-to-date food composition data. Local food composition databases were scored 2, as these represent best available local food composition data. Food composition databases borrowed from other countries were scored 1, as for trans fat food composition can vary from country to country.

[4] Studies of larger sample size (>1000) were scored 2, while data with <1000 participants were scored 1.

References Table S1.

1. Biro G, Hulshof KF, Ovesen L, et al. Selection of methodology to assess food intake. Eur J Clin Nutr **2002.** 56, Suppl. 2, S25–S32.
2. Nelson M. Methods and validity of dietary assessment. In Human Nutrition and Dietetics, 10th ed., 2001, pp. 311–329 [JS Garrow, WPT James and A Ralph, editors]. Edinburgh: Churchill Livingstone.
3. Willett W. Nutritional Epidemiology, 2nd ed. 1998, New York: Oxford University Press.

Table S2. Mean trans fat and saturated fat composition[1] of biscuits in 17 countries.

Country	Sampling year	Sampling method	Description biscuits	Sample size (analytical replicates) N (n)	Trans fat mean	Trans fat SD	Saturated fat mean g/100g fat	Saturated fat SD	Trans + Saturated fat mean	Intake data? [2]
Argentina [1]	2015	Selected local bakeries	Unpacked biscuits	12 (2)	11.1	9.5	45.6	10.0	56.7	No
Brazil [2]	2002/03	Major manufacturers	Cream cracker biscuits	5 (3)	19.9	9.1	24.1	9.0	44.0	2008/09
Brazil [3]	2012	Popular among students	Sweet biscuits	11 (3)	0.3	0.3	54.2	11.2	54.4	
China [4]	2006	Bestselling brands	Biscuits, crackers, wafers	18 (2)	2.8	6.3	57.4	10.6	60.2	2011
Germany [5]	2007/09	Not described	Biscuits and cookies mixture	85	1.1	2.2	62.5	11.9	63.6	2013
Italy [6]	2004	Bestselling brands	Biscuits, mixture	29	1.7	1.7	50.6	4.1	52.3	1995/96
India [7]	2009/11	Selected by availability	Biscuits, cookies, cream biscuits	33 (3)	5.4	3.2	58.3	16.4	63.7	No
Iran [8]	2011	Bestselling brands	Cream biscuits and simple biscuits	14	24.5	6.1	33.6	6.2	58.1	2004
Korea [9]	2005	Not described	Biscuits, cookies, crackers	14 (2)	6.0	6.8	53.7	10.0	59.7	No
Korea [9]	2008	Not described	Biscuits, cookies, crackers	14 (2)	2.0	1.6	59.5	6.2	61.5	
Lebanon [10]	2006	Bestselling brands	Biscuits and wafers	14 (2)	11.2	8.6	54.4	5.6	65.6	2006
Malaysia [11]	2004/05	Major manufacturers	Biscuits	5 (3)	0.3	0.3	50.9	2.6	51.2	No
Malaysia [12]	2009	Selected by availability	Biscuits, packed and unpacked	11 (3)	1.8[3]	1.5	58.9	16.3	60.7	
Malaysia [13]	2011	Bestselling brands	Biscuits and cookies	53 (2)	0.5	0.9	57.2	8.5	57.7	
New Zealand [14]	2006	Major manufacturers	Biscuits and cakes	11 (2)	1.1	1.2	62.6	4.5	63.7	2008/09
Pakistan [15]	2007	Bestselling brands	Biscuits	12 (6)	26.7	7.6	42.1	2.5	68.8	No
Poland [16]	2009/10	Not described	Biscuits, crackers, wafers	96 (3)	2.7		52.2		54.9	2009/10
Portugal [17]	2012	Not described	Cookies and biscuits	50 (2)	1.1	3.8	53.0	15.2	54.1	1995/96
Serbia [18]	2007	Bestselling brands	Biscuits	10 (3)	25.1	15.2	32.5	18.4	57.5	No
Serbia [18]	2008	Bestselling brands	Biscuits	8 (3)	5.4	9.8	50.4	14.2	55.8	
Serbia [18]	2009	Bestselling brands	Biscuits	16 (3)	3.3	5.2	62.6	8.6	65.9	
Sweden [19]	2001	Selective sampling	Biscuits, cookies and wafers	10 (2)	9.3	8.8	48.8	18.7	58.0	2015
Sweden [19]	2007	Selective sampling	Biscuits, cookies and wafers	25 (2)	0.3	0.3	54.6	17.3	54.9	
Turkey [20]	2005/06	Bestselling brands	Biscuits, digestives, petit beurre	9 (2)	2.7	1.6	48.6	2.3	51.3	No

[1] All measured by gas chromatography; [2] Trans fat intake data available, see Table 1 in main article; [3] One outlier was excluded from the dataset: one biscuit type contained 52g trans fat per 100g fat, when including this datapoint, trans fat content is 5.9 ± 14.4 g/100g fat.

References Table S2.

1. Negro, E., M.A. Gonzalez, C.A. Bernal, et al., Saturated and trans fatty acids content in unpackaged traditional bakery products in Santa Fe city, Argentina: nutrition labeling relevance. *Int J Food Sci Nutr*, **2016**: p. 1-7.
2. Martin, C.A., R. Carapelli, J.V. Visantainer, et al., Trans fatty acid content of Brazilian biscuits. *Food Chemistry*, **2005**. 93(3): p. 445-448.
3. Dias, F.D.S.L., M.E.A. Passos, M.D.G. Tavares Do Carmo, et al., Fatty acid profile of biscuits and salty snacks consumed by Brazilian college students. *Food Chemistry*, **2014**. 171: p. 351-355.
4. Fu, H., L. Yang, H. Yuan, et al., Assessment of trans fatty acids content in popular western-style products in China. *Journal of Food Science*, **2008**. 73(8): p. S383-S391.
5. Kuhnt, K., M. Baehr, C. Rohrer, et al., Trans fatty acid isomers and the trans-9/trans-11 index in fat containing foods. *Eur J Lipid Sci Technol*, **2011**. 113(10): p. 1281-1292.
6. Caponio, F., C. Summo, D. Delcuratolo, et al., Quality of the lipid fraction of Italian biscuits. *Journal of the Science of Food and Agriculture*, **2006**. 86(3): p. 356-361.
7. Kala, A.L.A., Cis-, trans- and saturated fatty acids in selected hydrogenated and refined vegetable oils in the indian market. *JAOCS*, **2012**. 89(10): p. 1813-1821.
8. Nazari, B., S. Asgary, and L. Azadbakht, Fatty acid analysis of Iranian junk food, dairy, and bakery products: Special attention to trans-fats. *Journal of Research in Medical Sciences*, **2012**. 17(10): p. 952-957.
9. Adhikari, P., F. Yu, J.H. Lee, et al., Comparative study of trans fatty acid content in 2005 and 2008 processed foods from Korean market. *Food Science and Biotechnology*, **2010**. 19(2): p. 335-341.
10. Saadeh, C., I. Toufeili, M. Zuheir Habbal, et al., Fatty acid composition including trans-fatty acids in selected cereal-based baked snacks from Lebanon. *Journal of Food Composition and Analysis*, **2015**. 41: p. 81-85.
11. Neo, Y.P., C.H. Tan, and A. Ariffin, Fatty acid composition of five malaysian biscuits (cream crackers) with special reference to trans- fatty acids. *International Food Research Journal*, **2007**. 14(3): p. 197-204.
12. Norhayati, M., A. Azrina, M.E. Norhaizan, et al., Trans fatty acids content of biscuits commercially available in Malaysian market and comparison with other countries. *International Food Research Journal*, **2011**. 18(3).
13. Norhayati, M.K., M.N. Mohd Fairulnizal, A. Zaiton, et al., Nutritional composition of selected commercial biscuits in Malaysia. *Sains Malaysiana*, **2015**. 44(4): p. 581-591.
14. Saunders, D., S. Jones, G.J. Devane, et al., Trans fatty acids in the New Zealand food supply. *Journal of Food Composition and Analysis*, **2008**. 21(4): p. 320-325.
15. Kandhro, A., S.T.H. Sherazi, S.A. Mahesar, et al., Monitoring of fat content, free fatty acid and fatty acid profile including trans fat in pakistani biscuits. *JAOCS*, **2008**. 85(11): p. 1057-1061.
16. Zbikowska, A., J. Rutkowska, and M. Kowalska, Consumption Safety of Pastries, Confectioneries, and Potato Products as Related to Fat Content. *J Am Coll Nutr*, **2015**. 34(6): p. 507-14.
17. Santos, L.A.T., R. Cruz, and S. Casal, Trans fatty acids in commercial cookies and biscuits: An update of Portuguese market. *Food Control*, **2015**. 47: p. 141-146.
18. Kravić, S.Z., Z.J. Suturović, J.V. Švarc-Gajić, et al., Fatty acid composition including trans-isomers of Serbian biscuits. *Hemijska Industrija*, **2011**. 65(2): p. 139-146.
19. Trattner, S., W. Becker, S. Wretling, et al., Fatty acid composition of Swedish bakery products, with emphasis on trans-fatty acids. *Food Chemistry*, **2015**. 175: p. 423-430.
20. Karabulut, I., Fatty acid composition of frequently consumed foods in Turkey with special emphasis on trans fatty acids. *International Journal of Food Sciences and Nutrition*, **2007**. 58(8): p. 619-628.

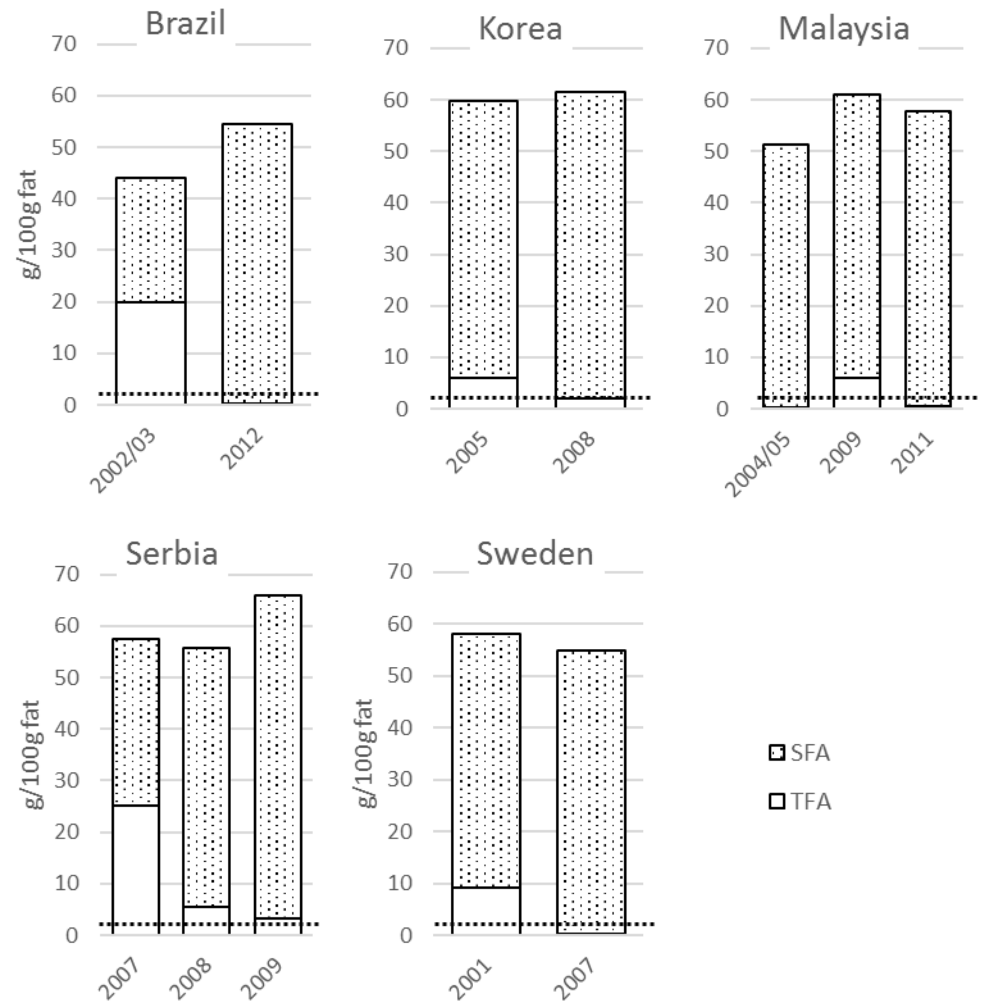

Figure S2. Time trends of mean trans and saturated fat content of biscuits in 5 countries. Data reported by year of sampling.
